# Detection of the mesenchymal-to-epithelial transition of invasive non-small cell lung cancer cells by their membrane undulation spectra†

**DOI:** 10.1039/d0ra06255c

**Published:** 2020-08-14

**Authors:** T. H. Hui, X. Shao, D. W. Au, W. C. Cho, Y. Lin

**Affiliations:** Department of Electrical and Electronic Engineering, The University of Hong Kong Hong Kong SAR China; Department of Mechanical Engineering, The University of Hong Kong Hong Kong SAR China ylin@hku.hk; HKU-Shenzhen Institute of Research and Innovation (HKU-SIRI) Shenzhen Guangdong China; Department of Clinical Oncology, Queen Elizabeth Hospital Hong Kong SAR China chocs@ha.org.hk

## Abstract

A cancer cell changes its state from being epithelial- to mesenchymal-like in a dynamic manner during tumor progression. For example, it is well known that mesenchymal-to-epithelial transition (MET) is essential for cancer cells to regain the capability of seeding on and then invading secondary/tertiary regions. However, there is no fast yet reliable method for detecting this transition. Here, we showed that membrane undulation of invasive cancer cells could be used as a novel marker for MET detection, both in invasive model cell lines and repopulated circulating tumor cells (rCTCs) from non-small cell lung cancer (NSCLC) patients. Specifically, using atomic force microscopy (AFM), it was found that the surface oscillation spectra of different cancer cells, after undergoing MET, all exhibited two distinct peaks from 0.001 to 0.007 Hz that are absent in the spectra before MET. In addition, by adopting the long short-term memory (LSTM) based recurrent neural network learning algorithm, we showed that the positions of recorded membrane undulation peaks can be used to predict the occurrence of MET in invasive NSCLC cells with high accuracy (>90% for model cell lines and >80% for rCTCs when benchmarking against the conventional bio-marker vimentin). These findings demonstrate the potential of our approach in achieving rapid MET detection with a much reduced cell sample size as well as quantifying changes in the mesenchymal level of tumor cells.

## Abstract 2

The membrane undulation spectra of cancer cells, measured by atomic force microscope, can be used to detect their transition from being mesenchymal- to epithelial-like.

## Graphical abstract

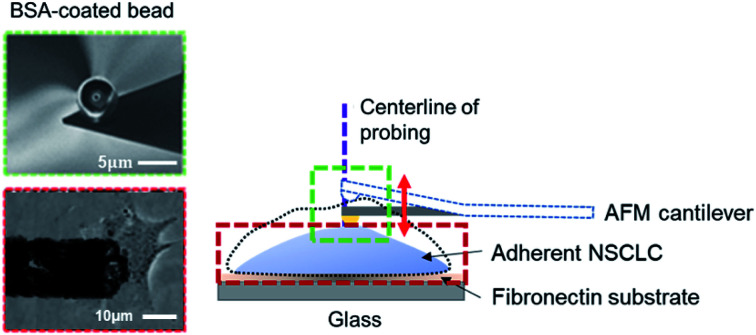

## Introduction

A major cause of the mortality of cancer patients is the spread of cancer from the original tumor site to distant organs or lymph nodes, a process referred to as distant metastasis.^1–3^ Mounting evidence has shown that the so-called mesenchymal-to-epithelial transition (MET) is essential for tumor cells to regain adhesion and migration capabilities, and eventually allows distant metastasis to take place.^4–6^ Given that the occurrence of MET in patients is very dynamic,^5^ a timely detection method for this transition could be critical for making prompt clinical decisions as well as the development of new intervention strategies.^7,8^ Currently, by monitoring signature molecules in the cell population, several methods including laser-scanning flow cytometry, immunostaining-fluorescence *in situ* hybridization (iFISH) and epithelial immunospot (EPISPOT)^9,10^ can be used to detect MET. However, a relatively large sample size and complicated multivariate analysis are often needed in these approaches.^11,12^ In addition, since the expression levels of most epithelial signature proteins can also be influenced by other biological processes,^13^ results from these methods alone may not be conclusive enough to capture the MET status. For this reason, tests of this kind often need to combine with other imaging techniques, such as CTs, PETs, and MRIs.^14–16^ Unfortunately, these imaging approaches may not be applicable to anatomical locations that are not accessible surgically or before the tumor has grown into certain size due to sensitivity, resolution and operational limitations.^17,18^

Interestingly, recent evidence has indicated that the invasiveness of tumor cells is intimately related to their physical characteristics. For example, it was found that invasive epithelial cells are generally softer, as well as more capable to undergo shape changes, than those with low metastatic potential^19–21^ both in cell lines and clinical samples from non-small cell carcinoma of the lung, breast ductal adenocarcinoma, and pancreatic adenocarcinoma*.*^22,23^ Along this direction, the use of deep learning method to relate the physical response of cells to different cancer biomarkers^11–13^ has received increasing attention, with the vision of achieving precision oncology eventually. These advances raise an interesting question: whether mechanical marker(s) can be established for MET detection as well as to overcome some of the aforementioned limitations associated with current methods.

In this study, we showed that membrane undulation characteristics of non-small cell lung cancer (NSCLC) cells could be used as an effective marker for MET detection, both in invasive model cell lines and circulating tumor cells (CTCs) from NSCLC cancer patients. Specifically, using atomic force microscopy (a powerful tool widely used in characterizing the properties of cells per biological samples^24–26^), we have found that the oscillation spectra of different NSCLC cells, after undergoing MET, all exhibited two distinct peaks in the range of 0.001–0.007 Hz that are absent before MET. Furthermore, by constructing neural network from the long short-term memory (LSTM) algorithm for learning, we showed that the position of these oscillation peaks can be used to predict the level of vimentin, a conventional mesenchymal marker, in NSCLC cells with high accuracy (>90% for cell lines and >80% for CTCs), highlighting the potential of our approach in achieving rapid and accurate MET detection in the future.

## Materials and methods

### Cell surface undulation measurement

**Fig. 1:**
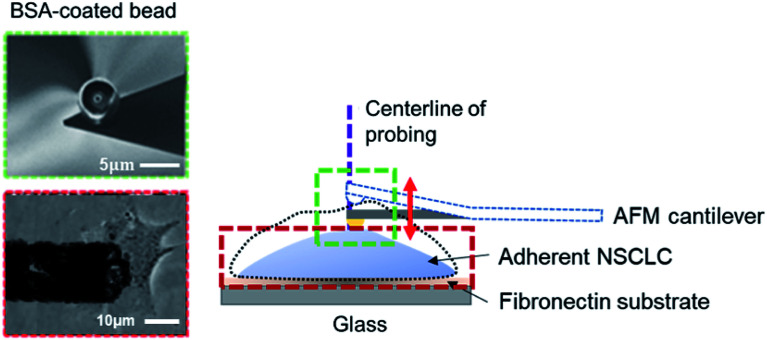
Illustration of the experimental setup. Adherent non-small cell lung cancer (NSCLC) cells were seeded on the glass-fibronectin substrate (see Materials and methods). A cantilever with a 5 μm bovine serum albumin-coated polystyrene bead (shown in the scanning electron microscopy (SEM) image in the inset, with a scale bar representing 5 μm) was used to monitor surface undulation at the cell center (defined as the intersection between the long and short axes of the cell). Note that, height change of the cell was traced by the vertical deflection, or equivalently the contact force, of the AFM cantilever, refer to the inset (scale bar = 10 μm).

An atomic force microscope (JPK NanoWizard II AFM, Veeco, USA), mounted onto a Zeiss AxioObserver with a fluid-cell-mounted cantilever (Microlevers, Veeco), was used to monitor the surface undulation of cells. The piezo platform (JPK Instruments) and photodiode signal were controlled by JPK NanoWizard II software (JPK Instrumental). A silicon nitride cantilever (NanoSensors) with a spring constant of 0.01 N m^−1^ was used in our experiments. Specifically, a spherical AFM tip (with a diameter of 200 nm) was first moved onto the cell surface at roughly the center of cell body and then held there for 4 h (refer to Fig. 1). During this period, drugs were added to the culture medium (see ESI A and C†) to trigger the MET of cells while the corresponding height variations of the cell (manifested as changes in the cantilever deflection/contact force) were recorded by the AFM. All tests were conducted outside the incubator at a temperature of 37 °C. For the calculation of the oscillation spectra and peak detection, see ESI B.†

### BSA coating of the AFM probe

To eliminate non-specific interactions between the cell and AFM probe, both the AFM cantilever and the spherical bead/tip were washed in nanopure water (Barnstead, 18.1 MΩ cm) and cleaned in UV for 30 min prior to silanization in phosphate buffered saline (PBS). The AFM probe was rinsed by nanopure water again before immersing into 50 μL Bovine Serum Albumin (BSA) (1 mg mL^−1^) for overnight incubation at 4 °C. After that, the probe was washed with PBS three times to remove excess BSA prior to the experiment.

### Coating on the cell culture dish

To immobilize cells on the culture dish for AFM measurement, 4 μg mL^−1^ of fibonectin in PBS was dispersed evenly on the dish surface by gentle aspiration. Cells (including different NSCLC cell lines and rCTCs) were then seeded on the dish at least 1 hour before the addition of MET inducers and subsequent membrane/surface undulation measurement.

### Culturing of repopulated circulating tumor cells (rCTCs)

CTCs were extracted by white cell isolation using Ficoll™-Paque Plus reagent (GE Healthcare, USA) from 15 mL of whole blood and then immunomagnetic separation method was used for spitting the CD45 positive cells for CTC enrichment (refer to ESI D†). After enrichment, CTCs were cultured in agarose hydrogel-based 3-D cell cultures, prepared with the CytoSelect Clonogenic Tumour Cell Isolation Kit (Cell Biolabs, Inc., San Diego, CA, USA) according to the manufacturer's instructions. In particular, the wells of a 24-well microplate were coated with base agar matrix and incubated at 37 °C for 10 min for gelation. Spheroids, containing extracted CTCs, were harvested for 8 days and then dissociated with the 3D Cell Culture Harvesting Kit (PromoKine, Heidelberg, Germany) for subsequent tests.

### Model cell line establishment and MET induction method

Different NSCLC cell lines (including HCC827 with EGFR deletion & amplification; NCI-H3255 with EGFR L858R; and NCI-H1975) were treated with 4 μg mL^−1^ of TNF-alpha and TGF-beta every 2 days to induce corresponding mesenchymal model cell lines (*i.e.*, after 0-, 4-, 8-, and, 12-d of treatment). For rCTCs, these chemicals were added within 1 d of their sub-culturing (following the protocols mentioned above). Both epithelial and mesenchymal markers (including beta-catenin, vimentin, and E-cadherin), cell viability, and Boyden chamber assay measurements were carried out to verify the invasiveness and stability of the model cell lines (see Fig. A1, A2, A3 and A4 in ESI A for details†). After that, the established mesenchymal model cells were treated with bufalin and three other potential MET inducers (*i.e.* rapamycin, *gamma*-secretase and *gamma*-tocotrienol, refer to ESI C for details†) and splitted into two halves, one for membrane undulation measurement and the other for western blotting to determine the change of mesenchymal levels (refer to ESI A†). rCTCs were treated with bufalin at the prescribed concentration stated in ESI C.†

### The long short-term memory (LSTM) based recurrent neural network learning model

Frequency spectra obtained from the recorded membrane undulation of HCC827, NCI-H1975, NCI-H3255 and CTC samples, along with the measured percentage changes of vimentin level in these cells, were inputted into the long-short term memory (LSTM) algorithm for iterative learning. After that, a neural network was constructed to recurrently learn the features from training data (*i.e.* from the LSTM block) first and then use test data to output the predicted mesenchymal level. Here, the LSTM block was established with a learning feedback linked to the output to reinforce the accuracy of the predicted vimentin concentration (as shown in Fig. 4A and see ESI E†). The weights and bias used in the network were determined by using Keras and TensorFlow 2.0 library iteratively from the measured vimentin levels in the training data.

## Results

As mentioned above, we pulsed three NSCLC cell lines (HCC827, NCI-H1975 and NCI-H3255, see ESI A†) with 4 μg mL^−1^ of TNF-alpha and TGF-beta (1 : 1 v/v)^27,28^ every 2 days to establish mesenchymal cell line models before triggering their MET. After eight passages, increased levels of different invasive markers (ESI A†) were observed from western blotting. Furthermore, such elevated expression level remained in cells even after 16 d, indicating that these cells have transited from the epithelial (less invasive) to the mesenchymal (highly invasive) state.

**Fig. 2:**
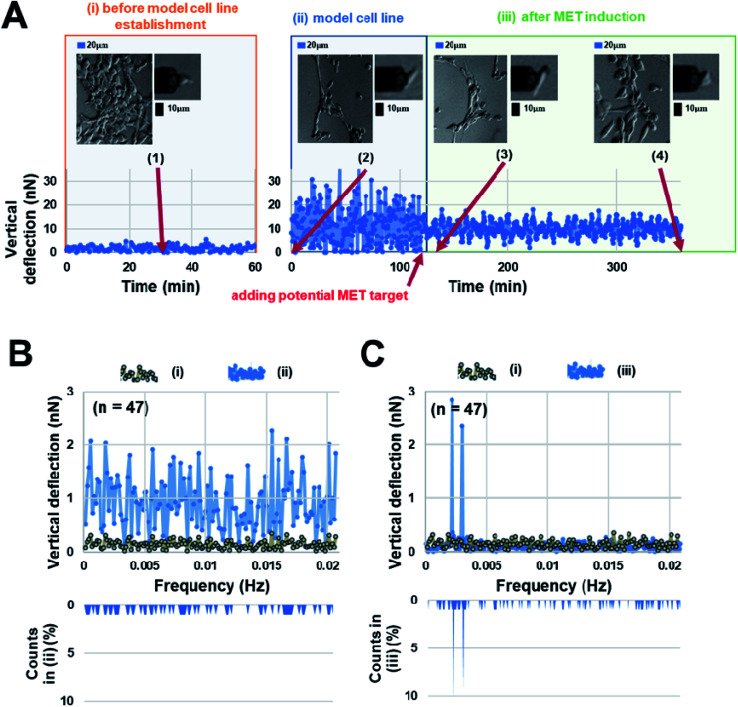
(A) A representative surface undulation profile of HCC827 cell line over time (i) before the establishment of the invasive model cell line; (ii) after model cell line establishment, and (iii) after MET induction. Representative morphologies of the cell at different stages are given in the insets. Corresponding oscillation spectra are shown in (B) and (C) respectively. Clearly, two oscillation peaks emerged after MET induction.

**Fig. 3:**
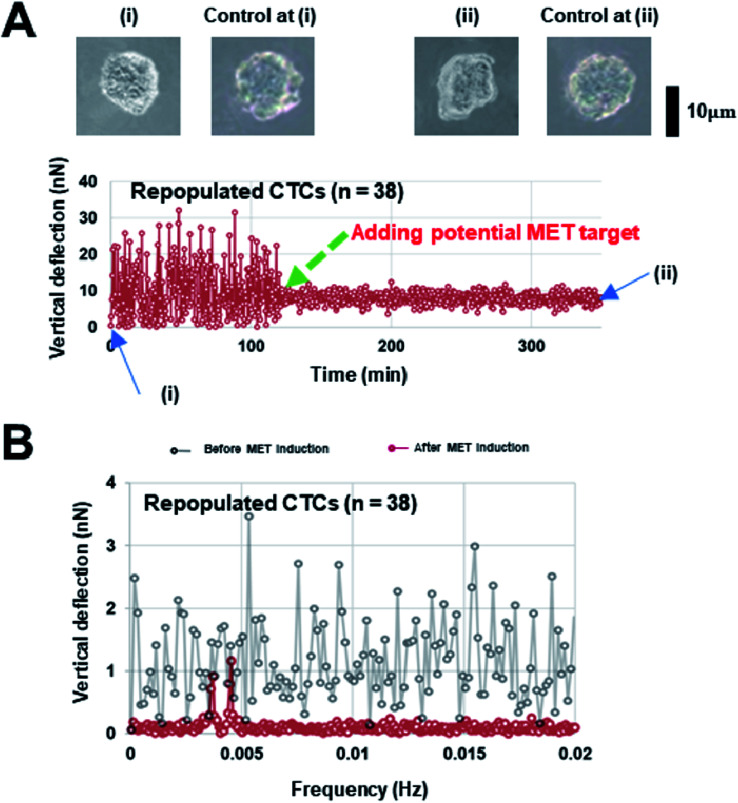
(A) Representative surface undulation profile and (B) the oscillation spectrum of a repopulated circulating tumor cell (rCTC), (i) represents the CTC before MET induction and (ii) corresponds to the CTC after MET induction. Cells remain circular in control (without MET induction), while a change in the cell morphology was observed after MET induction, refer to the insets.

Interestingly, our AFM measurement results showed that the magnitude of surface undulations of these three NSCLC cells in the mesenchymal state is much larger than that in the epithelial state (Fig. 2A), but no obvious peaks were observed in the corresponding oscillation spectra in the epithelial state (Fig. 2B). In comparison, once MET was induced (as confirmed by flow cytometry analysis, refer to ESI B and C for details†) in these cells, two distinct peaks, in the range of 0.001–0.007 Hz, emerged in the spectra (Fig. 2C). We then repeated the same experiment on repopulated circulating tumor cells (rCTCs) extracted from lung cancer patients (see ESI D† and Materials and methods) and found essential the same result (*i.e.* two peaks became visible in the oscillation spectra of rCTCs after MET), refer to Fig. 3A and B. In addition, it was also observed that, compared to the typical cuboidal morphology of parental NSCLC cell lines, the established model cell lines often assumed an elongated mesenchymal shape (Fig. 2A), which in conjunction with the increased surface undulation amplitude, showing that the membrane of cells has become much more active after acquiring invasiveness.

**Fig. 4:**
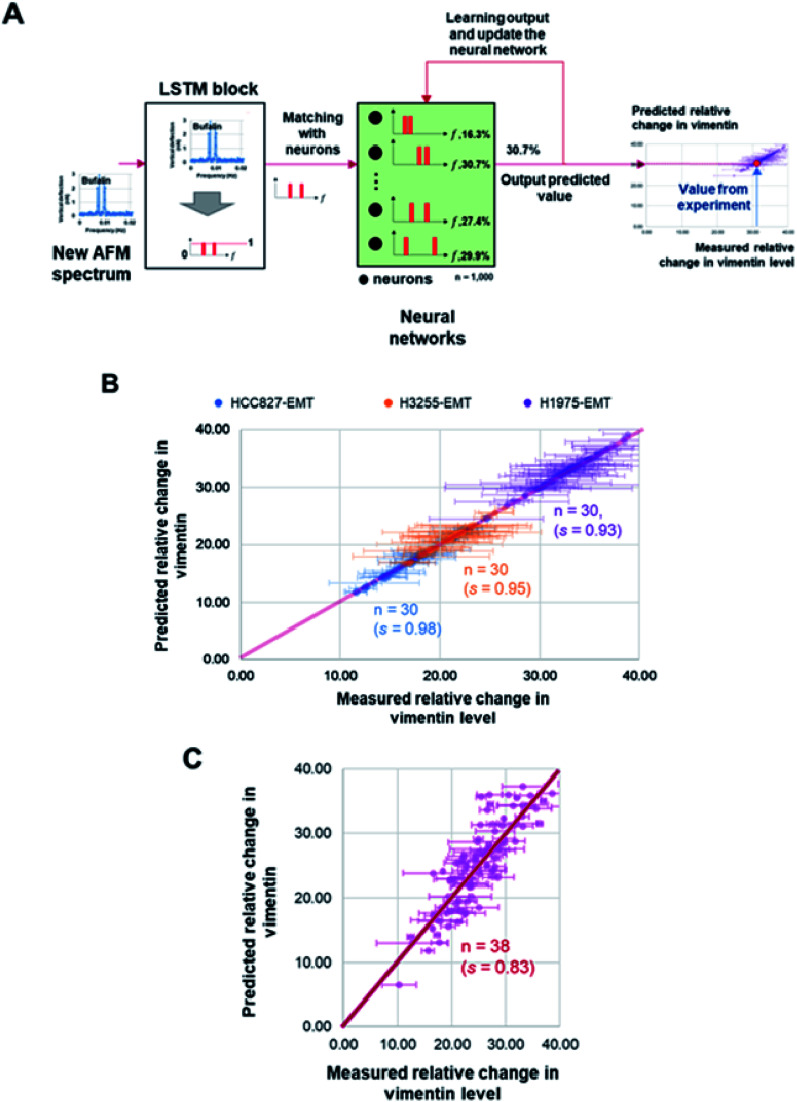
(A) Illustration of the long short-term memory (LSTM) based recurrent neural network learning algorithm. The recorded oscillation spectra were input to the LSTM block for learning. The peak frequency positions and vimentin levels were stored in the neural network. After comparison, corresponding vimentin level can be predicted from the matched neuron value. The predicted value will be feedbacked to the neural network for learning and updates in each neurons which will then be used for next matching. (B) Comparison between predictions from the proposed learning model and measured changes in the vimentin level (percentage change in the measured GAPDH) of three model NSCLC cell lines after MET induction. (C) Comparison between predictions from the model and measured changes in the vimentin level of repopulated circulating tumor cells (rCTCs) after MET induction. Here, s represents the *r*^2^ value between the prediction and measurement data.

To check whether the surface undulation characteristics discussed above can be used in MET detection, we also measured the vimentin (a signature protein for conventional MET/EMT detection) level in different cells (and at different states) and then utilized the LSTM model, as the learning block, to update the frequency positions stored in a neural network through matching/forgetting the value from the trained vimentin levels (Fig. 4A), refer to ESI E for details.† In total, 1000 sets of two-hour spectra within a six-hour windows of measurements from the three NSCLC model cell lines (HCC827, NCI-H1975 and NCI-H3255) after MET induction and corresponding changes in the vimentin level of the same batch were used in training. After that, another 30 recorded oscillation spectra of each model cell lines, along with 38 recorded rCTCs spectra, were input into the LSTM model where the expected vimentin levels were predicted. Comparisons among these predictions and the measured expression levels of vimentin are shown in Fig. 4B and C. Essentially, a prediction accuracy higher than 90% (or >80%) for model NSCLC cell lines (or rCTCs) has been achieved, demonstrating the potential of this approach in future prognosis of lung cancer.

## Discussion

In this study, we showed that the membrane undulation spectra of different lung cancer cells, after undergoing MET, all exhibit two distinct peaks in roughly the same frequency range (*i.e.* between 0.001 and 0.007 Hz) that are absent in the spectra before MET. In addition, by adopting deep learning (*i.e.* using the long short-term memory algorithm along with properly constructed neural network), we showed that the frequency position of the oscillation spectrum peaks can be used to predict the level of vimentin, a mesenchymal marker, in invasive NSCLC cells with high accuracy (>90% for model cell lines and >80% for rCTCs). Comparing to conventional methods such as laser-scanning flow cytometry (usually requiring tens of thousands of cells, refer to ESI C†), our approach can achieve rapid MET detection from a smaller sample size. In addition, the employed algorithm only uses the peak positions of the recorded surface undulation spectra and the measured vimentin level as the training input and therefore avoids multivariate features that may introduce noise/prediction errors. Given the highly dynamic nature of MET in cancer patients, our method may provide a rapid way to capture such transition and hence assist the making of prompt clinical decision in the future.

It must be pointed out that we connected the membrane undulation of tumor cells with the expression level of vimentin because (a) such intermediate filament is commonly used as an EMT/MET marker,^29–31^ and (b) accumulating evidences have also shown that vimentin itself plays a functional and developmental role in lung adenocarcinoma and metastasis.^30–32^ In addition, our results also indicated that different vimentin levels in three model cell lines developed from HCC827, NCI-H3255, and NCI-H1975 seem to be correlated with their slightly different membrane undulation characteristics (see Fig. B1† for example). Given that these cell lines have distinct molecular characteristics (HCC827 with EGFR deletion & amplification; NCI-H3255 with EGFR L858R; and NCI-H1975 with EGFR T790M and L858R respectively^15,33,34^), these observations suggest that the method may also be used to sort/differentiate cells according to their molecular characteristics and hence provide clues for the design of proper drug/gene treatment strategies.^35,36^

Finally, it is worth noting that the characteristic time corresponding to the frequency peaks identified here is ∼2–15 minutes, similar to that for vimentin remodeling^37^ and the formation of integrin-based cell-matrix adhesion.^38,39^ This suggests that the observed surface undulation of tumor cells may be caused by associated cytoskeleton and adhesion dynamics inside.^40–42^ On the other hand, recent evidence has also demonstrated that slow changes in the cellular volume can be triggered by active cross-membrane transport of water and ion molecules,^19,43–46^ which could also lead to the surface undulation of cells. Further investigations are certainly warranted to reveal the actual cause of the observed membrane oscillation period here.

## Author contributions

T. H. H., Y. L., W. C. C. devised this study. T. H. H. performed the experiments with assistance from X. S.; T. H. performed LSTM model training and construct the prediction model. D. W. A. prepared CTCs samples for the experiments. T. H. H., W. C. C., and Y. L. analyzed the results. T. H. H., W. C. C. and Y. L. prepared the manuscript. Y. L. supervised the study.

## Conflicts of interest

The authors declare that they have no competing interests.

## Supplementary Material

RA-010-D0RA06255C-s001
