# Supplementary material for: Detection of the mesenchymal-to-epithelial transition of invasive non-small cell lung cancer cells by their membrane undulation spectra
Source: RSC Adv. 2020 Aug 14;10(50):29999–30006. doi: 10.1039/d0ra06255c (PMC9056320; doi:10.1039/d0ra06255c)
Supplement: RA-010-D0RA06255C-s001 [file RA-010-D0RA06255C-s001.pdf]

Electronic Supplementary Material (ESI) for RSC Advances.
This journal is © The Royal Society of Chemistry 2020

Supplementary Materials

A. Establishing and verification of invasive model cell lines

Three human lung adenocarcinoma NSCLC cell lines (HCC827, NCI-H1975, and NCI-H3255) were obtained from the American Type Culture Collection (ATCC). Cells were cultured in RPMI1640 medium (Gibco) supplemented with 10% fetal bovine serum (FBS; Gibco) and 100-units/ml penicillin/streptomycin and incubated at 37°C under 5% CO_2 until 80% of confluency has been achieved in T75 flask (Nunc), monitored by optical microscopy with 100x magnification.

Next, to change these adenocarcinoma cell lines from epithelial to mesenchymal nature, 4ug/mL of TNF-alpha and 4ug/mL of TGF-beta (volume ratio 1:1 in cell culture medium) were added to the culture medium on day 2 and 4 during the six-day period. From western blotting results, it can be seen that all three adenocarcinoma cell lines showed increase of mesenchymal markers, including vimentin, N-cadherin, and a decrease of epithelial markers, beta-catenin and E-cadherin, after six days of treatment while the control marker GAPDH remaining unchanged.

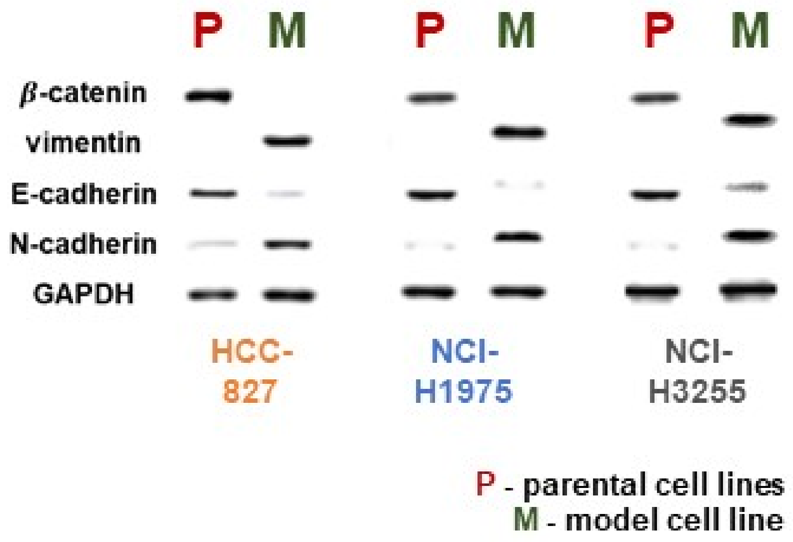

Figure A1 - Western blotting showing the effect of TNF-alpha and TGF-beta treatment on adherent non-small cell lung cancer cell lines HCC827, NCI-H1975, and NCI-H3255. The cells were pulsed with 4ug/mL of TNF-alpha and 4ug/mL of TGF-beta (volume ratio 1:1) two times (i.e. on day 2 and 4). After that, cells were spitted into two portions, with 2/3 (in volume) for continued culturing and 1/3 for Western blot. During Western blotting, cells were lysed in lysis

buffer (Thermofisher) containing phosphatase inhibitor cocktail C (Sigma-Aldrich) for total protein extraction. The concentration of proteins in cell lysates was quantified by Pierce BCA Protein Assay (Pierce Biotechnology, Inc., MA, USA) where 30 µg of proteins were loaded in each lane. Proteins were then separated on 12% SDS-PAGE and transferred to PVDF membranes using the iBlot® Dry Blotting System. The membranes were blocked in 5% skim milk in TBST (25 mM Tris–HCl, pH 7.4, 125 mM NaCl, 0.1% Tween 20) for 1 h, and subsequently incubated with appropriate primary antibodies at 4 °C overnight. After that, membranes were washed three times (with a duration of 10 min each) with TBST and then incubated with rabbit-conjugated secondary antibodies (Abcam, including beta-catenin, vimentin, E-cadherin, N-cadherin, and GAPDH) for 2 h at room temperature. The immune complexes formed were detected by chemiluminescence (SuperSignal™). Band quantification via densitometry was performed using ImageJ.

To verify that cells have indeed changed from epithelial to mesenchymal state and maintained the stability using this induction protocol, we performed a time-point measurement over 16 d, i.e. after 0, 1, 2, 4, 6, 8, 10, 12, 14 and 16 days of the induction treatment. Specifically, the 1/3 cells mentioned earlier were further spitted into 3 equal portions (upon cell passaging and topping up by adding 6 mL of culturing medium) for Western blotting, cell viability, and Boyden chamber assay tests to measure the invasiveness, stability and proliferation capability during and after the development of the model cell lines. As shown Figs. A2, A3, and A4, even after eight passages (16 d), the model cell lines showed stable mesenchymal properties, i.e. with increased expression level of mesenchymal markers, including vimentin, N-cadherin, and decreased epithelial markers like beta-catenin and E-cadherin. In addition, a cell viability between 70-90% was maintained (Fig. A3).

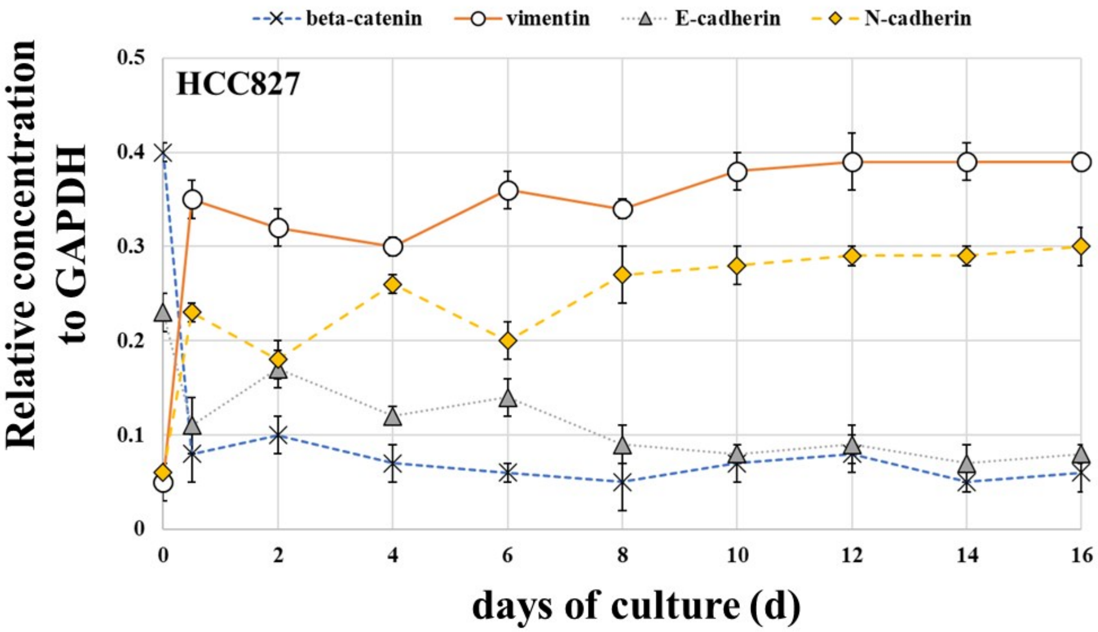
beta-catenin
vimentin
E-cadherin
N-cadherin
HCC827
Relative concentration to GAPDH
days of culture (d)

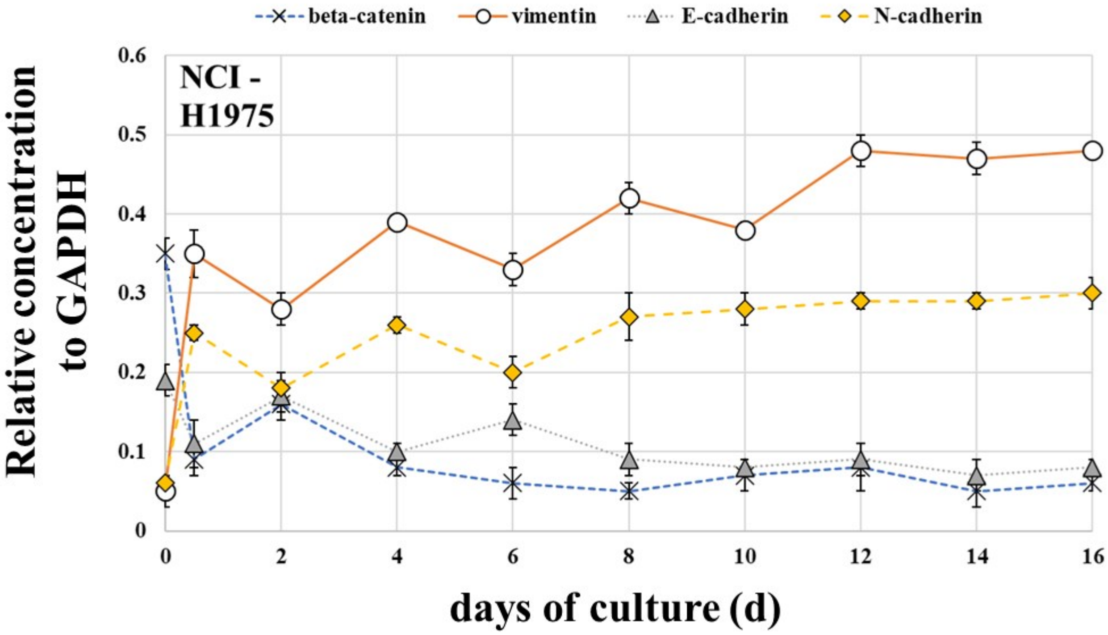
beta-catenin
vimentin
E-cadherin
N-cadherin
NCI - H1975
Relative concentration to GAPDH
days of culture (d)

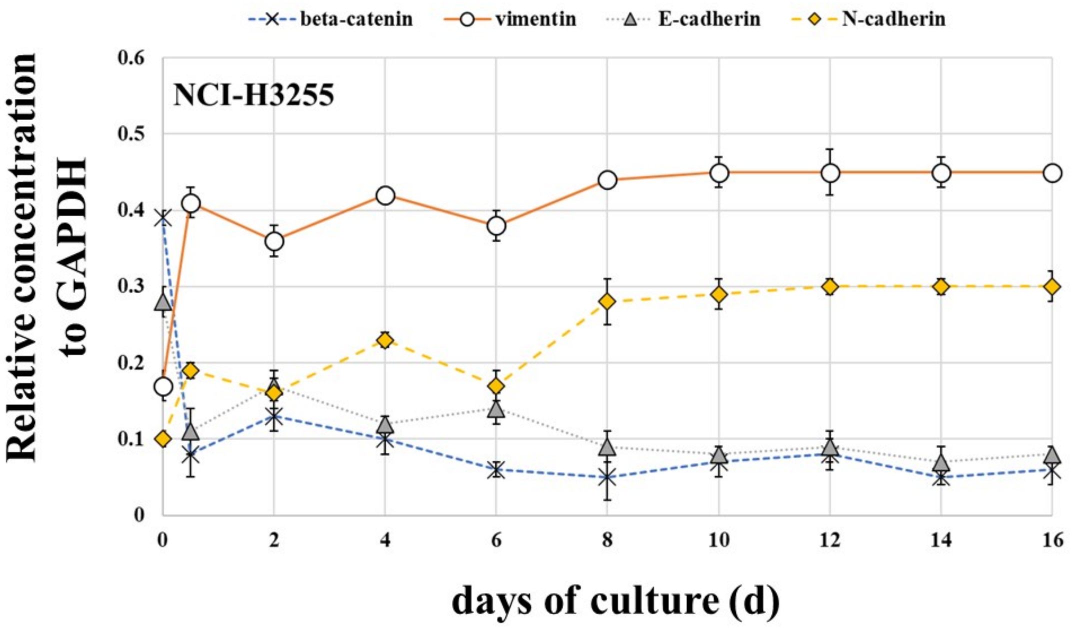

Figure A2 – Measured expression levels of different markers, including beta-catenin, vimentin, E-cadherin, in HCC827, NCI-H1975, and NCI-H3255 cell lines during the 16 d of culturing. Ten measurements were conducted by spitting the cell culture at 0, 1, 2, 4, 6, 8, 10, 12, 14 and 16 d, following the methods described in Figure A1. Error bar shows the standard error of the mean of each measurement. The TNF-alpha/TGF-beta treatments were performed on day 0-, 2-, 4-, 8-, and 12 of culturing.

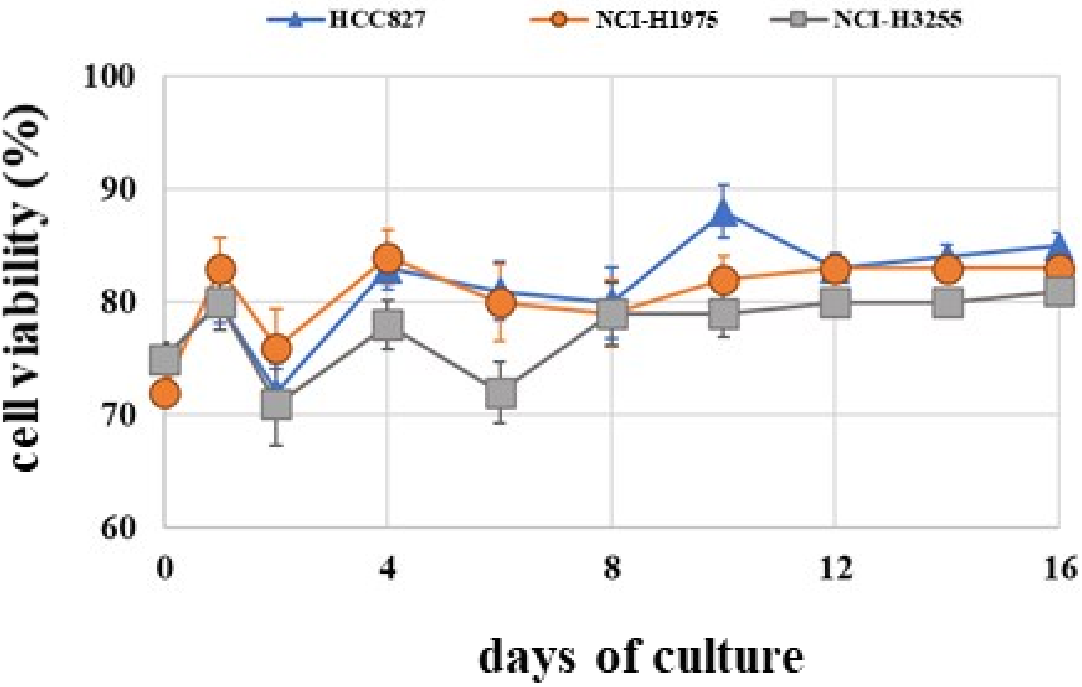

Figure A3 – viability changes of HCC827, NCI-H1975, and NCI-H3255 cells. For each passage (every 2 days), Annexin V surface expression (BD Biosciences) and caspase-3, 7 cleavage (Vibrant FAM kit; Life Technologies) were measured by Flow Cytometry (BD FACSCantoII Analyzer) as readouts for apoptosis. The percentage of non-apoptotic cells over the total cell population was defined as viability. Five experiments were carried out at day 0, 1, 2, 4, 6, 8, 10, 12, 14 and 16 where around 100,000 cells were measured. The error bar shows the standard deviation of measurement.

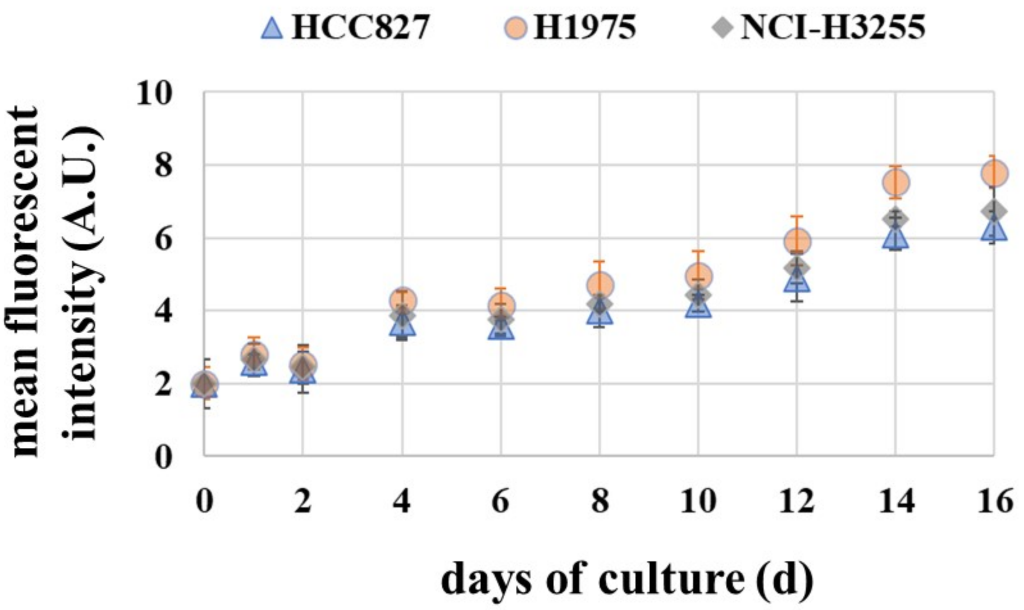

Figure A4 – change in the invasiveness of HCC827, NCI-H1975, and NCI-H3255 cells from Boyden chamber assay. Five measurements were taken at each time point. Error bar shows the standard error of the mean of each measurement. During such test, transwell inserts (8.0 μm membrane, Corning) were placed in 6-well plates and covered with 1 mL of Matrigel (Sigma-Aldrich) diluted in phenol-red-free RPMI1640 at a final concentration of 2 mg/mL to form the Boyden chamber assay. The inserts were incubated at 37°C and 5% CO2 for 2 h to allow gelation. A total of 1×10^5 cells were seeded in the insert with 1.5 mL phenol-red-free RPMI1640 solution. The bottom of wells was filled with 2 mL phenol-red-free RPMI1640 solution with FBS (10%) as a chemoattractant. Cells were incubated at 37°C and 5% CO_2 for 24 h. After that, the Matrigel was removed by three washes with phosphate buffer saline. Cells that migrated through the porous membrane (indicated as invasive cell here) were harvested with 1 mL PBS-EDTA (1 mM, Thermo Fisher Scientific) and permeabilized by 0.1% Triton X-100 (Sigma). Calcein AM (Sigma) was dissolved in Dimethylsulfoxide (DMSO), with gentle agitation performed every 15 min, to stain of the gel membrane. Fluorescence was measured using a microplate fluorometer (Gemini). The excitation and emission wavelengths were set to be 485 and 538 nm, respectively.

B. Obtaining the frequency spectrum

The undulation spectra of cell membrane were obtained by discrete Fourier transform, that is

$$F(\omega) = \sum_{t=-\infty}^{\infty} f(t) e^{-j\omega t}$$

, where t is time, ω denotes the frequency, $j = \sqrt{-1}$ and f is the measured temporal profile of membrane undulation. Representative undulation spectra of HCC827, NCI-H1975, and NCI-H3255 model cell lines after MET (triggered by different MET inducers) are shown in Fig. B1.

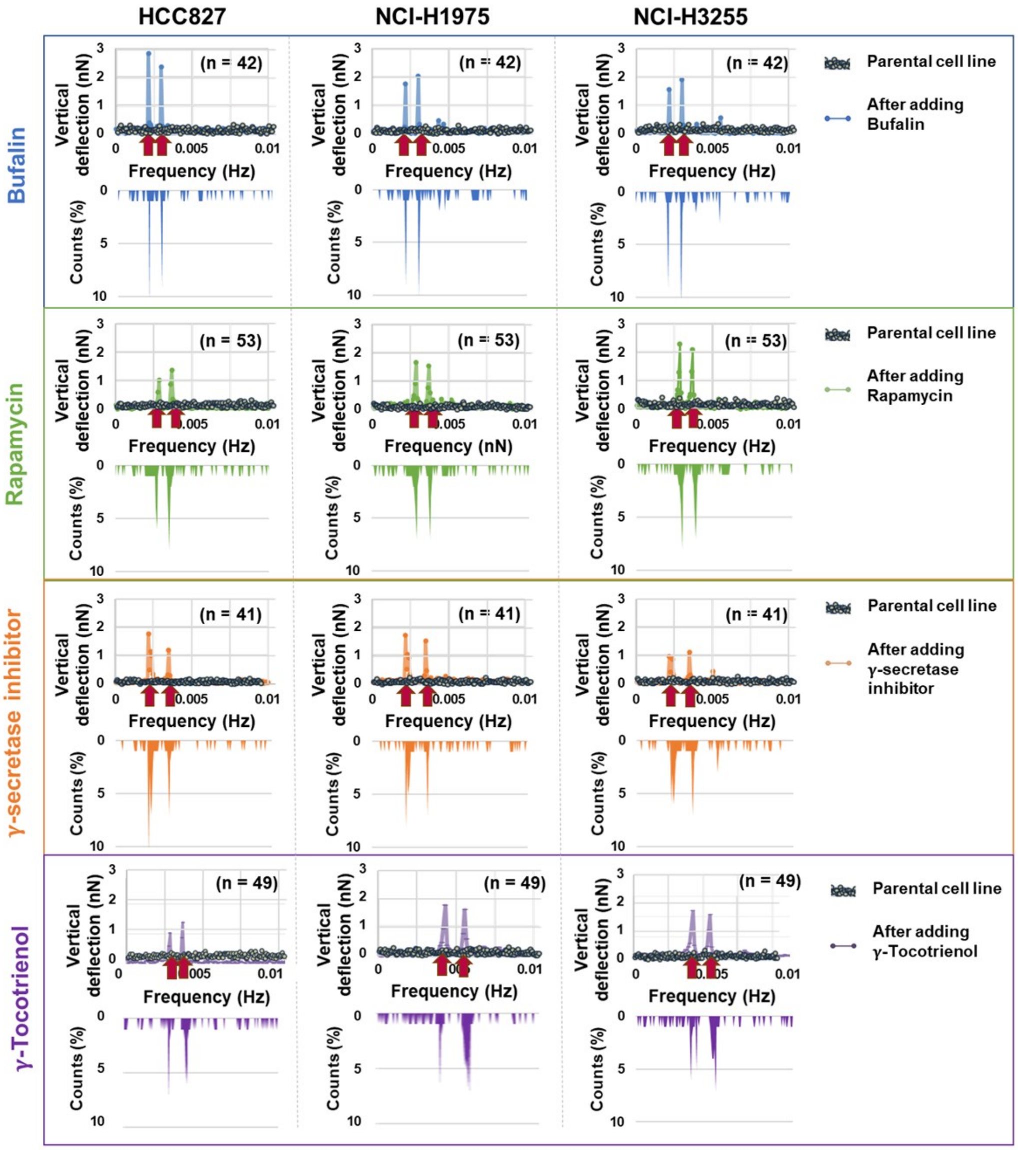

Figure B1 – Averaged membrane undulation spectra of HCC827, NCI-H1975, and NCI-H3255 model cell lines before and after MET (triggered by different MET inducers). Clearly, two peaks emerge after MET each case whose position can be detected by quadratic interpolation of sinusoidal spectrum analysis [1]. Here n represents the number of cells measured.

C. MET induction method and validations

Potential MET inducers, including 6.5 nM bufalin (Sigma-Aldrich) [2-4], 10 ng/mL rapamycin (Sigma-Aldrich) [5, 6], 8 μM γ-secretase inhibitor (Sigma-Aldrich) [7, 8], and 50 μM γ-tocotrienol [9, 10],

were added during AFM surface undulation measurement. The expression level of beta-catenin, vimentin, E-cadherin, and N-cadherin were monitored at different times points for the same cell batch (under the influence of different inducers). The results shown here concluded 1) the epithelial cell lines can be induced into mesenchymal nature from the methods stated in Supplementary A; and 2) the potential MET inducers above can force the induced cell lines with the mesenchymal nature back into epithelial nature, refer to Fig. C1.

(1) parental
(2) after model cell line establishment
(3) 10-min after MET induction
(4) 120-min after MET induction
(5) 240-min after MET induction

		HCC827				NCI-H1975				NCI-H3255			
		β-catenin	vimentin	E-cadherin	N-cadherin	β-catenin	vimentin	E-cadherin	N-cadherin	β-catenin	vimentin	E-cadherin	N-cadherin
Bufalin	(1)	++	--	++	--	++	--	++	--	++	--	++	--
	(2)	--	++	-	++	--	++	--	++	--	++	--	++
	(3)	+	+	+	+	+	+	-	-	+	+	-	-
	(4)	++	--	++	--	++	--	++	--	++	--	++	--
	(5)	++	--	++	--	++	--	++	--	++	--	++	--
Rapamycin	(1)	++	--	++	--	++	--	++	--	++	--	++	--
	(2)	--	++	--	++	--	++	--	++	--	++	--	++
	(3)	+	+	-	-	-	-	-	-	-	-	-	-
	(4)	++	--	++	--	++	--	++	--	++	--	++	--
	(5)	++	--	++	--	++	--	++	--	++	--	++	--
γ-secretase inhibitor	(1)	++	--	++	--	++	--	++	--	++	--	++	--
	(2)	--	++	--	++	--	++	--	++	--	++	--	++
	(3)	+	+	-	-	+	-	-	-	+	+	-	-
	(4)	++	--	++	--	++	--	++	--	++	--	++	--
	(5)	++	--	++	--	++	--	++	--	++	--	++	--
γ-Tocotrienol	(1)	++	--	++	--	++	--	++	--	++	--	++	--
	(2)	--	++	--	++	--	++	--	++	--	++	--	++
	(3)	+	+	+	--	+	+	+	--	+	+	-	-
	(4)	++	--	++	--	++	--	++	--	++	--	++	--
	(5)	++	--	++	--	++	--	++	--	++	--	++	--

++ : relative expression > 0.2;
+ : 0.1 < relative expression < 0.2;
- : 0.05 < relative expression < 0.1;
-- : relative expression < 0.05

Figure C1 – expression level changes of different markers, including beta-catenin, vimentin, E-cadherin, and N-cadherin, in HCC827, NCI-H1975, and NCI-H3255 model cell lines before and

10, 120 and 240 min after MET induction using Bufalin, Rapamycin, gamma-secretase inhibitor, and gamma-tocotrienol. For reference, marker levels in parental cell lines (i.e. before the establishment of invasive model cell lines) are also shown.

Laser-scanning flow cytometry was also used to confirm and detect the MET in our invasive model cell lines. Specifically, before and after 2 h of MET induction, the established HCC827, NCI-1975 and NCI-H3255 model cell lines were dissociated using 0.05% tryspin/EDTA (Invitrogen) for 5 min, followed by removal of trypsin and washing with Phosphate Buffer Saline (PBS). After that, cells were labeled by E-cadherin (PerCP-Cy™5.5 Mouse Anti-Human CD324 (E-Cadherin), BD Bioscience USA) and N-cadherin (Alexa Fluor® 488 Mouse anti-Human CD325, BD Bioscience, USA) dyes following the manufacturer's protocols.

The fluorescent intensities of E- and N-cadherin of these cells, before and after MET induction, were measured and analyzed using BD FACS Canto II Analyzer. As illustrated in Figure C2, C3 and C4, the relative N-cadherin/E-cadherin concentration decreases/increases after treatment of different MET inducers, verifying that MET in these model cell lines has indeed occurred. Note that, the relative concentration was estimated by subtracting the background intensity from the measured intensity. In each case, 10,000 cells were measured.

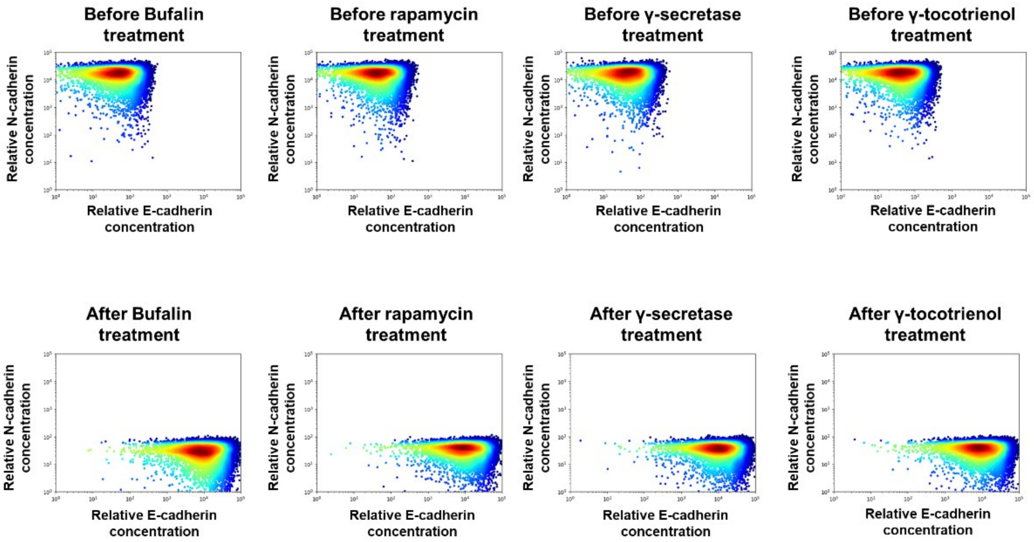

Figure C2 - Flow cytometry map of established HCC827 model cell line before and after MET induction.

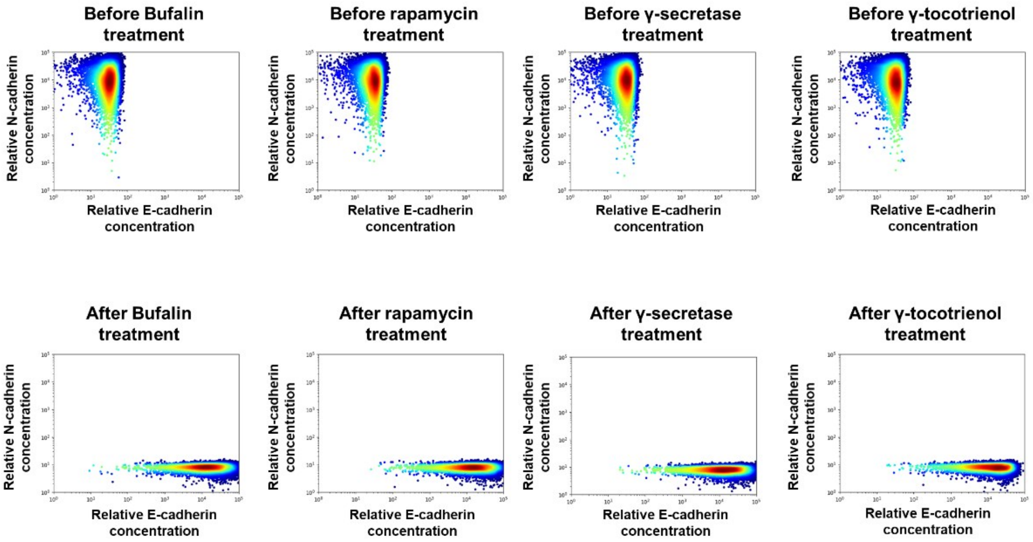

Figure C3 - Flow cytometry map of established NCI-H1975 model cell line before and after MET induction.

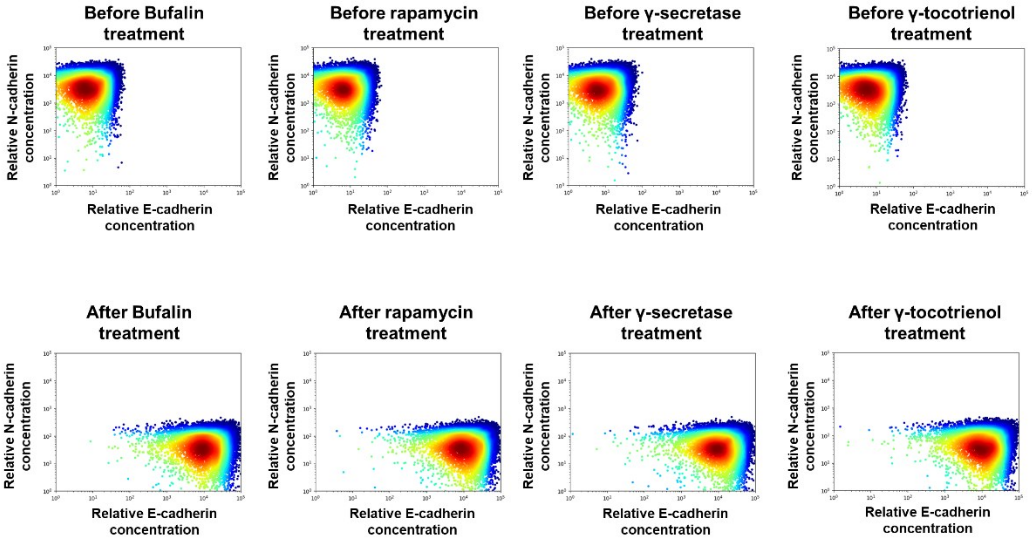

Figure C4 - Flow cytometry map of established NCI-H3255 model cell line before and after MET induction.

D. Clinical sample recruitment and isolation

CTCs were extracted by white cell isolation using ficoll™-Paque Plus reagent (GE Healthcare, USA) from 15 mL of whole blood followed by immunomagnetic separation method for spitting the CD45+ cells for CTC enrichment. The leukocyte layer was extracted and temporarily stored using Opti-MEM with reduced serum (reduced serum, phenol-red free, Gibco) as a buffer for ficoll™-Paque Plus reagent (GE Healthcare, USA). In particular, the CTCs were enriched using Dynabeads CD45 (Invitrogen™, Thermo Fisher Scientific, Inc.) placed on a magnet for 15 min at room temperature. Same medium was used for volume top-up, washing and resuspension following the manufacturer's protocol.

After enrichment, CTCs were cultured in agarose hydrogel-based 3-D cell cultures prepared with the CytoSelect Clonogenic Tumour Cell Isolation Kit (Cell Biolabs, Inc., San Diego, CA, USA) according to the manufacturer's instructions. In particular, wells of a 24-well microplate were coated with base agar matrix and incubated at 37°C for 10 min for gelation. Repopulated circulating tumor cells were then dissociated from the from the spheroids after 8 days of harvest (with 3D Cell Culture Harvesting Kit (PromoKine, Heidelberg, Germany) following the manufacturer's protocol) for subsequent membrane undulation test.

After the AFM experiment, cells were collected for multiparametric flow cytometry (BD FACSARia cell sorter) labeling with CTC markers, including Epithelial cell adhesion molecule (EpCAM), cytokeratin 8 (CK8), CK18, and CK19. In particular, cells were fixed by 4.7% formaldehyde and 0.1% Triton X-100, and then stained with the aforementioned antibodies. 405nm, 488nm, 633nm lasers were used for the analysis. The measured batch was discarded if either one of the markers exhibiting more than 15% of negative result from the total population.

E. Long short-term memory (LSTM) based recurrent neural network learning model

LSTM was used in the neural network to learn the AFM frequency spectrum and predict the vimentin level. The LSTM block contains three computation networks: the Forget network (Fig. E1), the Input Network (Fig. E2) and the Output Network (Figure E3).

As described in Fig. 4, training data containing 1,000 vimentin level (i.e. V_a with a =1, 2, …1000, each defined as a neuron) and the corresponding 1,000 frequency spectra, with a sampling interval of 0.5s, were input to the LSTM block. Each frequency spectrum was equally divided in frequency range, denoted as X_f^a where the subscript f varies from 1 to 14,440 (i.e. the number of frequency divisions).

For each set of training data (i.e. for each a), a Forget Network (Fig E1) was used to compute the "Forget Array" F_f^a comprising elements of 0 and 1 based on the training data X_f^a and the Previous Hidden State $H^a{}_f^{-1}$. The Forget Array was utilized in the next step as a gate to forget (filter out) certain information in the Previous Cell State $C^a{}_f^{-1}$. Next, the Input Network (Fig. E2) was used to calculate the Current Cell State C_f^a (Long Term Memory) based on the training data X_f^a, Previous Hidden State $H^a{}_f^{-1}$ and Previous Cell State C_f^{a-1}. Lastly, the Output Network was used to update the Current Hidden State H_f^a (Short Term Memory) based on training data, Previous Hidden State $H^a{}_f^{-1}$ and Current Cell State C_f^a. The process was repeated until the last set of training data.

After the training, each vimentin level V_a was matched with a Hidden State H_f^a. This allows us to make a prediction of the vimentin level for a given frequency spectrum, by means of calculating its corresponding Hidden State and then matching with the proper vimentin value.

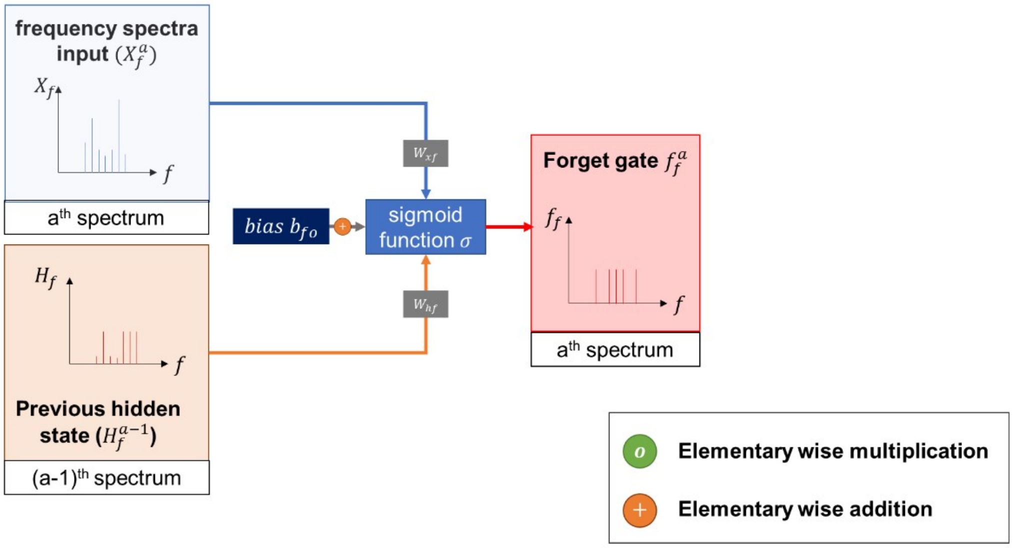

Figure E1 – Schematic showing the operational flow of the Forget Network f_f^a in the LSTM block.

The process of computing the Forget Array, f_f^a, is shown in Figure E1. Specifically, the previous hidden state H^{a-1}_f and X_f^a were multiplied elementarily with a pair of weights, W^a_{xf} and W^a_{hf}, and then added with a constant bias b_{fo}. A logic function σ, defined as $\sigma(y) = \left\lfloor \frac{1}{1+e^{-y}} \right\rfloor$ with $y = W^a_{xf}X_f^a + W^a_{hf}H^{a-1}_f + b_{fo}$, is then introduced to determine the value of each element in the Forget Array, that is

$$f_f^a = \sigma\left(W^a_{xf}X_f^a + W^a_{hf}H^{a-1}_f + b_{fo}\right). \qquad [S1]$$

Note that, depending on the value of y, $\frac{1}{1+e^{-y}}$ varies between zero and unit. The bracket operator $\lfloor\rfloor$ here rounds up its value to 0 or 1, i.e. the resulting Forget Array contains elements of 0 or 1 only. This array serves as an operator to keep or forget the next input information/data, with 1 referring to keep the information while 0 corresponding to forget the information.

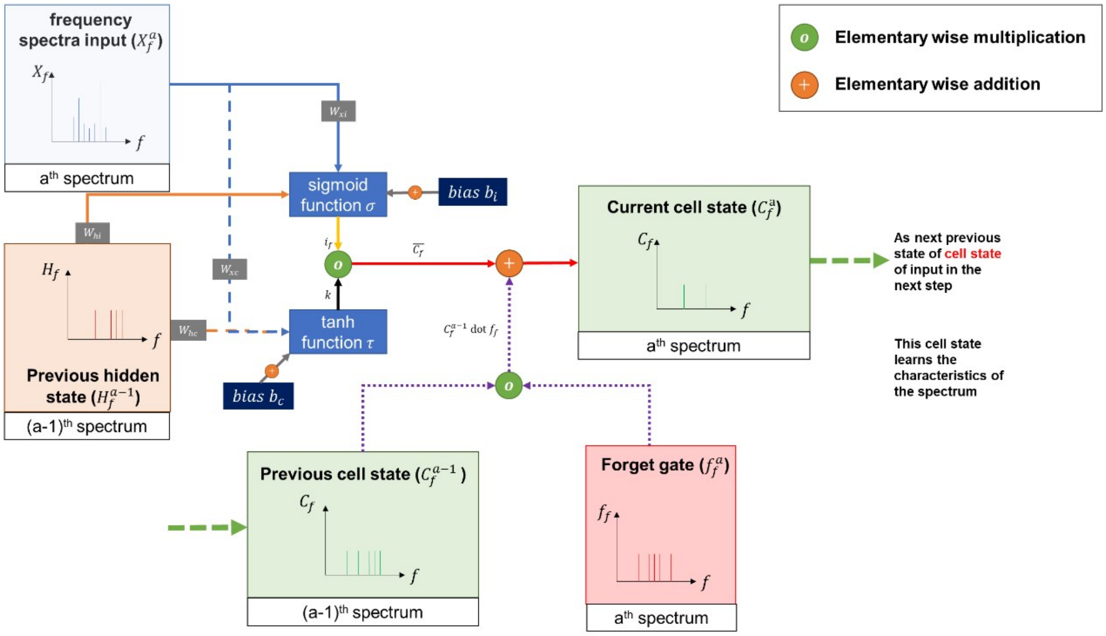

Figure E2 – Schematic showing the operational flow of the Input Network in the LSTM block.

The Input Network was used to calculate the Current Cell State C_f^a (Long Term Memory) based on the training data X_f^a, Previous Hidden State H_f^{a-1} and Previous Cell State C_f^{a-1}. First of all, the training data X_f^a and the Previous Hidden State H_f^{a-1} were converted into an intermediary array i_f^a containing element values between 0 and 1 by using a sigmoid function

$$i_f^a = \sigma(W_{xi}^a X_f^a + W_{hi}^a H_f^{a-1} + b_i) \qquad [S2]$$

where W_{xi}^a and W_{hi}^a are a pair of weights and b_i is a constant bias.

Next, to get the input cell for long term storage, similar operation was carried out to the training data X_f^a and the Previous Hidden State H_f^{a-1} with two different weights, W_{xc}^a and W_{hc}^a, and another bias b_c. After that, a hyperbolic tangent (τ) function was used to sustain the long range from

iterations before going to zero [11]. The output from τ allows increases and decreases in the cell state and the output can be positive and negative, ranging from -1 to +1 as

$$k = \tau\left(W_{xc}^{a}X_{f}^{a} + W_{hc}^{a}H^{a-1}_{f} + b_{c}\right). \qquad [S3]$$

To output the long-range result for storing purpose, a dot product of i_f^a and k was carried out with the value denoted as $\bar{C}_f^{\,a}$, i.e.

$$\bar{C}_f^{\,a} = i_f^a \; dot \; k. \qquad [S4]$$

After getting the long-range result as an input, $\bar{C}_f^{\,a}$ will pass the frequency position to the next iteration if the vimentin level matches with the neuron. Specifically, the previous cell state, C^{a-1}_{f} was multiplied with f_f^a by dot product and then added to the long-range current result $\bar{C}_f^{\,a}$ to yield the output of cell state, that is

$$C_f^a = \bar{C}_f^{\,a} + \left[C^{a-1}_{f} \; dot \; f_f^a\right]. \qquad [S5]$$

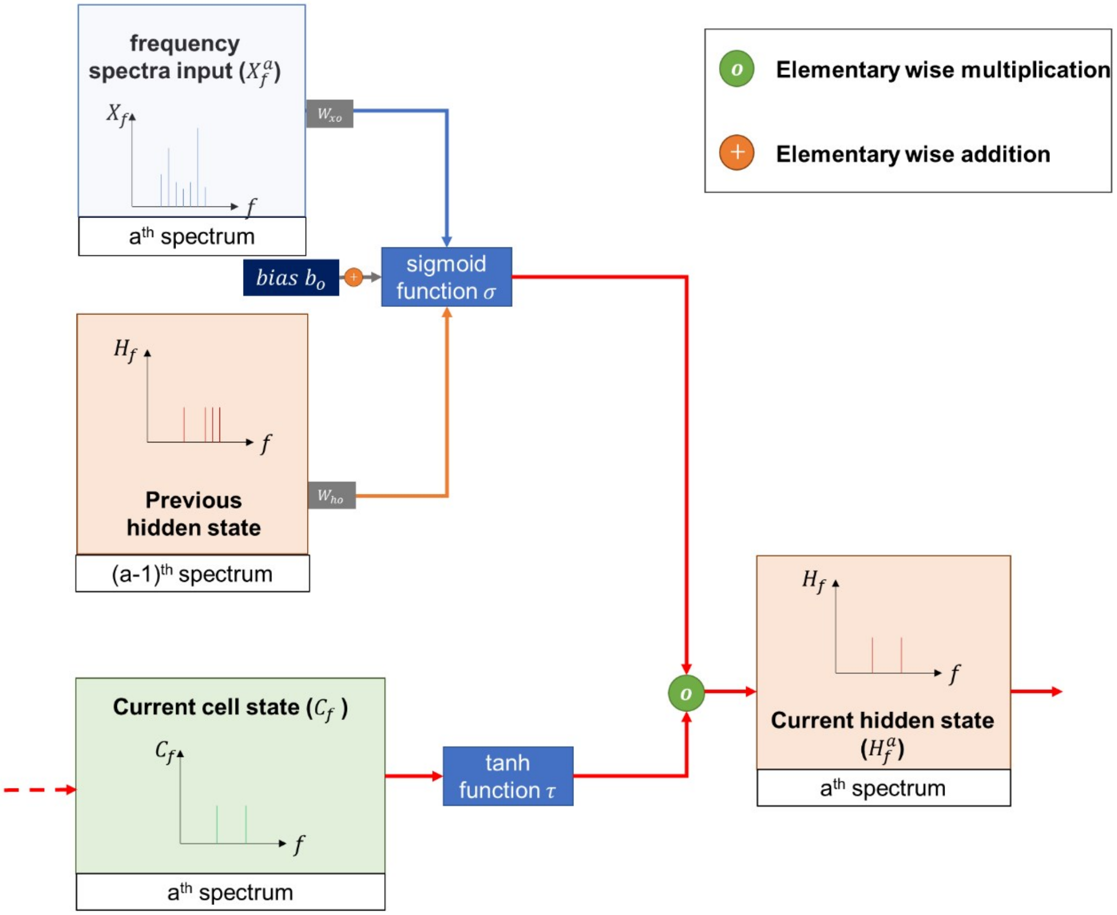

Figure E3 - Schematic showing the operational flow for the temporary output of each LSTM block and updating of the current hidden state

Finally, to update the hidden state H_f^a, the current cell state C_f^a is extended by a hyperbolic tangent function τ, and pointwise multiplying with the recurrent product $\sigma(W_{xo}^{a}X_f^a + W_{ho}^{a}H^{a-1}_{f} + b_o)$, i.e.

$$H_f^a = \sigma(W_{xo}^{a}X_f^a + W_{ho}^{a}H^{a-1}_{f} + b_o)\ dot\tau\left(C_f^a\right). \qquad [S6]$$

After training, the tested spectra were input one-by-one into the algorithm described above. In each case, the output spectrum was identified from the neurons trained and updated such spectrum in the same neuron, serving as a mechanism to learn the frequency in each neuron.

$W^a_{xi}, W^a_{hi}, W^a_{xf}, W^a_{hf}, W^a_{xc}, W^a_{hc}, W^a_{xo}$, and W^a_{ho} are the weights and b^a_i, b^a_{fo}, b^a_c *and* b^a_o, are the additive biases at the input gate, the forget gate, the cell state and the output gate, respectively. These weights and bias were assigned initial values randomly in the range of (-0.1 to 0.1) and then updated after each iteration by Adam optimization method with the Keras and TensorFlow libraries [12, 13], using a learning rate of 0.1 in each iteration he hidden and cell states were initialized by introducing random noise (0 to 1) with the same dimension of the frequency spectrum.

To validate the training data, cross-validation of training data was performed by k-fold cross-validation (k=10) [14]. Specifically, the training data (1000 AFM spectra peaks with 1000 expected changes in vimentin level) were divided into 10 bins of equal size randomly. After training, 30 additional data were used to test the training data. For each iteration, the predicted value was feedbacked to the cell state as the input and updated the hidden state for the next prediction. > 90% accuracy was achieved in cell lines and >80% was achieved in rCTC. The training efficiency (expressed as accuracy and loss against epochs, passing through all the blocks above) compared against other existing training methods is shown in Fig. E4. Our method is able to converge after only ~30 epochs, presumably because that both long- and short-term information were captured during the recurrent learning/training in the algorithm proposed here.

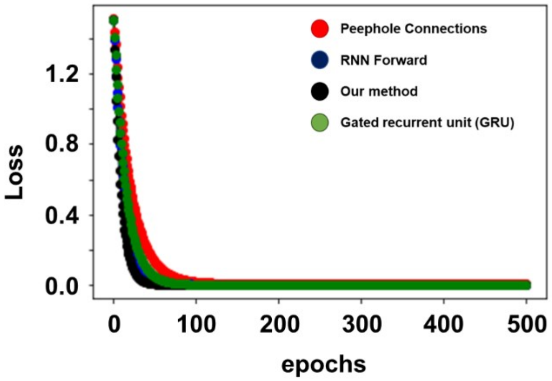

Figure E4 - Comparison of training efficiency and accuracy across iterations (epochs) between our method and conventional training algorithms including Peephole connections [11], vanilla RNN forward [15] and gated recurrent unit (GRU) [16]. Loss here represents the percentage difference between the predicted value and the measured value between two consecutive epochs.

References

1. P. Du, W. Kibbe and S. Lin, *Bioinformatics (Oxford, England)*, 2006, **22**, 2059-2065.
2. X. H. Kang, J. H. Zhang, Q. Q. Zhang, Y. H. Cui, Y. Wang, W. Z. Kou, Z. H. Miao, P. Lu, L. F. Wang, Z. Y. Xu and F. Cao, *Cell Physiol Biochem*, 2017, **41**, 2067-2076.
3. F. Cao, Y.-B. Gong, X.-H. Kang, Z.-H. Lu, Y. Wang, K.-L. Zhao, Z.-H. Miao, M.-J. Liao and Z.Y. Xu, *Toxicology and Applied Pharmacology*, 2019, **379**, 114662.
4. L. Chen, W. Mai, M. Chen, J. Hu, Z. Zhuo, X. Lei, L. Deng, J. Liu, N. Yao, M. Huang, Y. Peng, W. Ye and D. Zhang, *Pharmacological Research*, 2017, **123**, 130-142.
5. D. J. Boffa, F. Luan, D. Thomas, H. Yang, V. K. Sharma, M. Lagman and M. Suthanthiran, *Clin Cancer Res*, 2004, **10**, 293-300.
6. C. Rolfo, E. Giovannetti, D. S. Hong, T. Bivona, L. E. Raez, G. Bronte, L. Buffoni, N. Reguart, E. S. Santos, P. Germonpre, M. Taron, F. Passiglia, J. P. Van Meerbeeck, A. Russo, M. Peeters, I. Gil-Bazo, P. Pauwels and R. Rosell, *Cancer Treat Rev*, 2014, **40**, 990-1004.
7. J. Tsurutani, K. A. West, J. Sayyah, J. J. Gills and P. A. Dennis, *Cancer Res*, 2005, **65**, 8423-8432.
8. J. Konishi, K. S. Kawaguchi, H. Vo, N. Haruki, A. Gonzalez, D. P. Carbone and T. P. Dang, *Cancer Res*, 2007, **67**, 8051-8057.
9. S. Wada, Y. Satomi, M. Murakoshi, N. Noguchi, T. Yoshikawa and H. Nishino, *Cancer Lett*, 2005, **229**, 181-191.
10. P. Zarogoulidis, A. Cheva, K. Zarampouka, H. Huang, C. Li, Y. Huang, N. Katsikogiannis and K. Zarogoulidis, *J Thorac Dis*, 2013, **5**, 349-352.
11. F. Gers, N. Schraudolph and J. Schmidhuber, *Journal of Machine Learning Research*, 2002, **3**, 115-143.
12. D. Kingma and J. Ba, *International Conference on Learning Representations*, 2014.
13. A. Sherstinsky, *Physica D: Nonlinear Phenomena*, 2020, **404**, 132306.
14. J. Rodríguez, A. Pérez and J. A. Lozano, *Pattern Analysis and Machine Intelligence, IEEE Transactions on*, 2010, **32**, 569-575.
15. N. Léonard, S. Waghmare and Y. Wang, *arXiv.org*, 2015, 1511.07889
16. K. Cho, B. van Merriënboer, C. Gulcehre, F. Bougares, H. Schwenk and Y. Bengio, *arXiv.org*, 2014, 1406.1078
